# Enhancement of Air-Entrained Grout-Enriched Vibrated Cemented Sand, Gravel and Rock (GECSGR) for Improving Frost and Thawing Resistance in CSGR Dams

**DOI:** 10.3390/ma18010155

**Published:** 2025-01-02

**Authors:** Wambley Adomako Baah, Jinsheng Jia, Cuiying Zheng, Baozhen Jia, Yue Wang, Yangfeng Wu

**Affiliations:** 1 State Key Laboratory of Simulation and Regulation of Water Cycle in River Basin, China Institute of Water Resources and Hydropower Research (IWHR), Beijing 100038, China; jiajsh@iwhr.com (J.J.); zhengcy@iwhr.com (C.Z.); jiabaozhen1987@sohu.com (B.J.); yue.wang@bjtu.edu.cn (Y.W.); 2 School of Civil Engineering, Beijing Jiaotong University, Beijing 100044, China; wuyangfeng_0211@tju.edu.cn; 3 State Key Laboratory of Hydraulic Engineering Simulation and Safety, Tianjin University, Tianjin 300072, China

**Keywords:** cemented sand, gravel and rock dam (CSGRD), Grout-Enriched Vibrated Cemented Sand, Gravel and Rock (GECSGR), CVC, GERCC, freeze–thaw resistance, admixture

## Abstract

Cemented Sand, Gravel, and Rock (CSGR) dams have traditionally used either Conventional Vibrated Concrete (CVC) or Grout-Enriched Roller Compacted Concrete (GERCC) for protective and seepage control layers in low- to medium-height dams. However, these methods are complex, prone to interference, and uneconomical due to significant differences in the expansion coefficient, elastic modulus, and hydration heat parameters among CSGR, CVC, and GERCC. This complexity complicates quality control during construction, leading to the development of Grout-Enriched Vibrated Cemented Sand, Gravel, and Rock (GECSGR) as an alternative. Despite its potential, GECSGR has limited use due to concerns about freeze–thaw resistance. This project addresses these concerns by developing an air-entrained GECSGR grout formulation and construction technique. The study follows a five-phase approach: mix proportioning of C_180_6 CSGR; optimization of the grout formulation; determination of grout addition rate; evaluation of small-scale lab samples of GECSGR; and field application. The results indicate that combining 8–12% of 223 kg/m^3^ cement grout with 2–2.23 kg/m^3^ of admixtures, mud content of 15%, a marsh time of 26–31 s. and a water/cement ratio of 0.5–0.6 with the C_180_6 parent CSGR mixture achieved a post-vibration in situ air content of 4–6%, excellent freeze–thaw resistance (F300: mass loss <5% or initial dynamic modulus ≥60%), and permeability resistance (W12: permeability coefficient of 0.13 × 10^−10^ m/s). The development of a 2-in-1 slurry addition and vibration equipment eliminated performance risks and enhanced efficiency in field applications, such as the conversion of the C_180_4 CSGR mixture into air-entrained GECSGR grade C9015W6F50 for the 2.76 km Qianwei protection dam. Economic analysis revealed that the unit cost of GECSGR production is 18.3% and 6.33% less than CVC and GERCC, respectively, marking a significant advancement in sustainable cement-based composite materials in the dam industry.

## 1. Introduction

Based on the concept of Hardfill Dam and Trapezoidal Cemented Sand and Gravel (CSG) Dam, the concept of Cemented Sand Gravel and Rock Dam (CSGRD), a new type of dam, was proposed by Jinsheng Jia of China in 2004 [1,2,3,4]. CSGRD is an economical and sustainable type of dam with a character between an embankment (soil-rock) dam and a gravity dam [2,3,4,5,6]. The technique further expands on the sizes of the local aggregates in trapezoidal CSG Dam by Japanese Engineers from 80 mm to 150 mm in dams and up to 300 mm in coffer dams with little or no screening increasing construction efficiency and saving cost relative to the former. Again, Cemented Artificial Sand, Gravel, and Rock (CASR), a subcategory of CSGRD, can be built in conditions for steep riverbeds or when local aggregates are not available and proven to be 10% more economical than CSGRD [1,7,8].

According to SL674-14, the technical guidelines for Cemented Granular Materials issued by the Ministry of Water Resources of the Peoples’ Republic of China, the CSGR material is produced by achieving certain shear strength through the minimal cementation of sand, gravel, and rock aggregates in their rightful proportions. Through that, the compressive strength classes C_180_4, C_180_6, C_180_8, and C_180_10 for the dam body and C_180_15 and C_180_20 for Rich-Mix and Rich Slurry CSGR, respectively, can be produced successfully to meet project requirements [8,9,10].

Unlike the Concrete dam, in which the body is designed to be watertight, the body of the CSGRD is similar to the Faced-Symmetrical Hardfill Dam (FSHD), and the CSG Dam is designed to provide structural support but has the potential to develop into seepage along its compacted layers just like Roller Compacted Concrete (RCC). Hence, it requires upstream protection and a watertight layer in conjunction with the grouting curtains, drain holes, cushion layers, etc., which control the seepage under the dam [11,12,13].

The FSHD by France, the CSG Dam by Japanese Engineers, and the CSGRD by Jinsheng Jia of China form the new classification known as HCC, with each letter representing a dam in the order mentioned above [5]. The FSHD considers the usage of precast concrete and geomembranes to construct the upstream watertight layer, whilst the CSG is known for using Conventional Vibrated Concrete (CVC) [5,14]. CSGRD has also previously employed either CVC or Grout-enriched Roller Compacted Concrete (GERCC) with Grout-enriched Vibrated CSGR (GECSGR) or Rich-slurry CSGR sandwiched between the former layer and body of the dam in medium-height dams [5,15].

However, the construction of this structure is complex and has potential interferences. This complexity arises due to notable differences between CSGR and CVC or GERCC in the expansion coefficient, elastic modulus, and hydration heat parameters. Consequently, ensuring quality control during construction is often a cause for concern [11,16,17].

Fortunately, in the search for methods to improve GECSGR, a previous study by Hazaree et al. (2011) demonstrated that it is possible to entrain air in standard Roller-Compacted Concrete (RCC) and achieve adequate freeze–thaw resistance. However, efforts by researchers and contractors to incorporate air-entraining admixtures into Grout-Enriched RCC (GERCC) to improve freeze–thaw resistance have been unsuccessful [18,19].

In addition, earlier research highlighted an early attempt at the Horseshoe Bend dam in New Zealand, where a freeze–thaw resistant facing was desired. To achieve this, the grout was heavily dosed with an air-entraining admixture to obtain 3–4% residual air in the GERCC facing. However, the initial trials failed because the grout became excessively foamy and could not penetrate the spread RCC layer [20].

Furthermore, one of the most detailed studies on air-entrained GERCC, involving laboratory tests and field trials, was reported in a previous study. The laboratory tests evaluated grout formulations, dosage rates, and methods for combining the grout with RCC. These tests revealed that achieving a homogeneous mixture by placing the grout on the top or bottom of the RCC is very challenging. Additionally, when internal vibrators were used to mix the grout and RCC, the air content, and thus the freeze–thaw resistance, significantly decreased. Similar results were observed in field trials and separate field trials conducted by Tatro et al. (2008). These findings underscore the challenge of using air-entrained GERCC; current construction techniques require substantial vibration to ensure a homogeneous mixture. However, this vibration removes much of the entrained air from the grout, reducing its freeze–thaw resistance [21,22,23].

According to Musselman et al. (2016), organic and synthetic air-entraining admixtures successfully achieved air contents between 15% and 25% at their maximum recommended dosages, with the synthetic admixture providing the most stable air content and superior freeze–thaw resistance. While the water-reducing admixture helped reduce bleed and permeability, it caused greater variability in air content, making it unsuitable for air-entrained grout-encapsulated RCC (GERCC). Water-repelling and efflorescence-controlling admixtures improved performance, with the saline-based version showing better freeze–thaw resistance, whereas the powder water-resisting admixture negatively affected air entrainment and was not recommended for GERCC [24].

Because the cons of the previously adopted materials outweighed the pros, research is needed to evaluate various construction techniques to determine their impact on grout distribution within the CSGR and freeze–thaw resistance of air-entrained GECSGR. Another reason was to identify an optimum placement technique and vibration level that balances these factors. Researchers of RCC therefore suggested future studies exploring these parameters for different RCC mixtures. Additionally, the development of automated processes for grout placement, mixing with RCC, and consolidating the mixture to enhance the quality and consistency of GERCC was recommended [5,24].

Jia et al. (2016), after laboratory investigations, put forward that Rich-Mix CSGR and GECSGR of permeability grade and Frost resistance up to W12 (equivalent to a permeability coefficient of 0.13 × 10^−3^ m/s) and F300 (300 freeze–thaw cycles with mass loss less than 5% and initial modulus of 60%), respectively, can be employed for frost/thawing resistance, anti-seepage control, and anti-carbonation zones in CSGRD based on the project’s actual requirement. A novel approach was suggested based on the experiences from previous CSGR projects and GERCC, which is incorporating vibrated grout-enriched CSGR in constructing the protection and seepage control layer of the low-height (h < 15 m) CSGR dam for the first time. In that work, a slurry addition rate of 8–12% was proposed and tested, and a new 2-in-1 grout addition and vibratory equipment were developed with a grouting capacity of 12 m^2^/h alongside a new surface water stop structure for the structural joints (i.e., T-shaped waterstop structure with boding strength and water-pressure resistance of 1.8 MPa and 1.6 MPa, respectively, for the layer). This reduced the cost of the layer to 50% relative to CVC and GERCC previously employed in CSGRD construction [1,5].

To build on the milestone of the GECSGR, the next goal of the project was to develop a grout mixture and construction method that enables the creation of air-entrained GECSGR in situ. This section of the research consists of five phases to systematically accomplish this goal: (i) development of mix proportion for parent CSGR; (ii) optimizing the grout mixture, including the type and quantity of chemical admixtures; (iii) assessing small-scale lab samples of CSGR and grout mixed in a laboratory mixer; (iv) evaluating large-scale lab samples of CSGR and grout combined using real-world construction methods; (v) carrying out field application. The findings shall be compared to the performances when CSGR and grout are mixed in a lab mixer, considering a strong freeze–thaw resistance. The caution is in field applications, as the level of vibration needs to be carefully managed to maintain performance using the 2-in-1 grouting and vibrating equipment to yield air-entraining and excellent field results [5,24].

**Figure 1:**
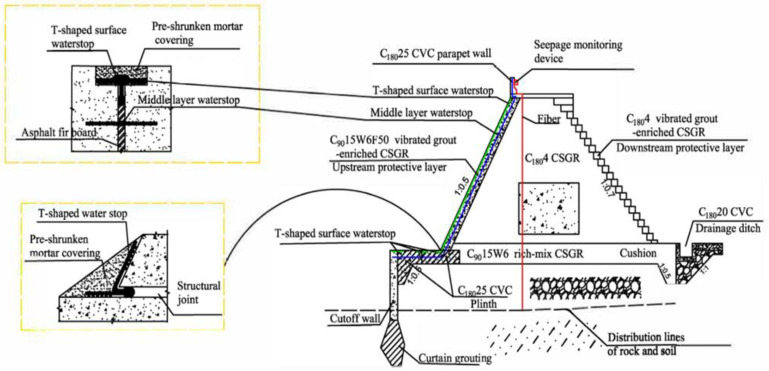
Cross-section of Qianwei CSGR Dike with GECSGR layer (courtesy of Wu et al., 2024) [5].

The Qianwei Navigation and Hydropower Hub Project is located in Sichuan Province, China, in the lower Minjiang River. It is part of a larger shipping and hydropower plan for the Leshan–Yibin region and is classified as a second-class, large-scale project. The project’s main objective is navigation while also supporting power generation, water supply, and irrigation for local development. The hub’s water level is 335 m, with a 228 million cubic meters reservoir capacity. A protection dam (a dike as shown in Figure 1) 2.76 km long and up to 15 m high is located 2 km upstream of the hub and is designed to withstand a 20-year flood. It is built on a 13 m thick layer of sand and gravel bed.

Initially, a geotextile membrane core wall and rockfill dam were planned, but concerns about leakage and overtopping led to an alternative design: a Cemented Sand, Gravel, and Rock (CSGR) dam with a trapezoidal cross-section. The upstream slope ratio is 1:0.5 and the downstream slope is 1:0.7. The dam’s main body has a 180-day compressive strength of 4 MPa (C_180_4) of CSGR material, while the upstream protective layer, designed to resist seepage, has a 90-day compressive strength of 15 MPa (C9015W6F50), along with impermeability of W6 (permeability coefficient of 0.42 × 10^−10^ m/s) and frost resistance grade of F50 (50 Freeze–thawing cycles). T-shaped surface water stops are used for joint sealing between the dam and its foundation, which is composed of sand and gravel with drainage capacity, eliminating the need for a separate drainage layer. This innovation and application of air-entrained GECSGR to the Qianwei Dike has enhanced construction efficiency significantly while simultaneously reducing project costs [1,5]. It is worth noticing that the results discussed in this project will provide a reference for constructing more sustainable and economical CSGR dams and hydraulic structures in the future.

## 2. Materials and Methods

### 2.1. Raw Materials

#### 2.1.1. Cement

The cement is P.O 42.5 ordinary Portland cement produced by Sichuan Qianwei Baoma Cement Co., Ltd., located in Leshan, Sichuan Province of China. The cement quality and chemical test results are listed in Table 1 and Table 2, respectively, and the cement mortar strength test results are shown in Table 3. It can be seen from both tables that the test results of the inspected properties meet the technical requirements specified in GB175-2020 “General Purpose Portland Cement” [25] (ASTM C150 equivalent) [26].

#### 2.1.2. Fly Ash

The fly ash is grade II ash provided by Leshan Runsen Waste Recycling Co., Ltd. in Leshan, a community in China. The physical and chemical characteristics are shown in Table 4 and Table 5, respectively. In Table 4, the fineness is determined by screening fly ash through a 45 µm square hole sieve, and the fineness of the fly ash samples is expressed by the mass percentage of the sieve residue on the sieve. The water demand ratio is the ratio of the water demand of CSGR materials prepared with fly ash compared to specimens without fly ash (control) (ASTM C618) [27]. The strength activity index was also obtained by the measurement of the compressive strength of the test mortar and the control mortar after 28 days, and finally, the activity of the test mortar was determined by the ratio of the compressive strength of the two. The tables show that the test results of the tested performance comply with GB/T1596-2017 [28]. Class II fly ash coal ash requirements (ASTM C618 equivalent) [27].

#### 2.1.3. Chemical Admixtures

The six admixtures including organic air-entraining admixture (OAEA), synthetic AEA (SAEA), polycarboxylate-based water-reducing agent (WR), latex-based water-repelling and efflorescence-controlling admixture (LW), saline-based efflorescence-reducing and water-repelling admixture (SW) and a powdered water-resisting and superplasticizing admixture (PW) in Table 6 were provided by Shijiazhuang Mayor An Yucai Building Materials Co., Ltd. in Shijiazhuang, China. The performance test was carried out using the cement in Table 1. In Table 3, the results indicate that the admixture from the company and cement exhibited good compatibility. Also, in Table 6, the difference in setting time refers to the difference between the setting time of the CSGR with admixture and the control (i.e., those without admixtures) [29]. The ratio of compressive strength refers to the ratio between the compressive strengths of samples with admixture to those without admixture at different ages [30]

#### 2.1.4. Natural Aggregates

**Figure 2:**
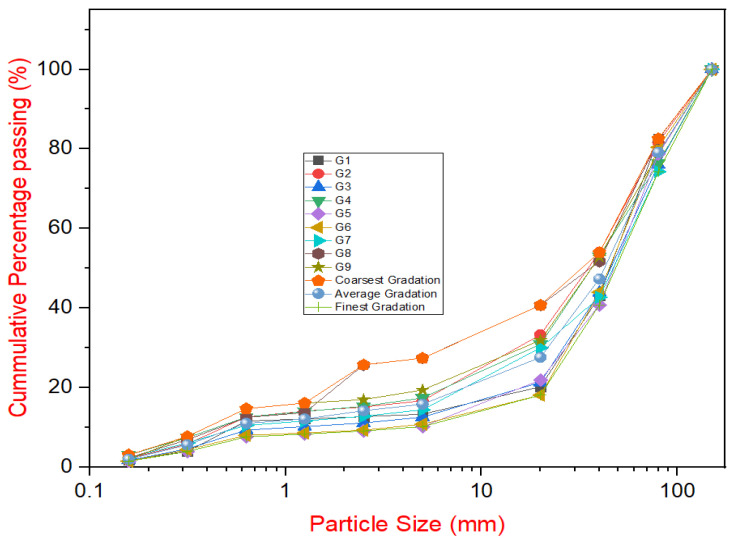
Particle size distribution curve of aggregates.

Riverbed sand, gravel, and rock aggregate from the upstream section of the Qianwei Navigation and Hydropower Hub Project were selected for particle screening tests. A total of 22 gradation tests were conducted on the aggregates, with particle sizes ranging from 150 to 80 mm, 80–40 mm, 40–20 mm, 20–5 mm, and below 5 mm. The gradation envelope for the gravel aggregate is shown in Figure 2, where G1 to G9 represent the different material gradations. For the composition of the gradation, the proportion of extra-large stones (80–150 mm) ranged between 16% and 32%, with an average of 22.3%. The proportion of large stones (40–80 mm) varied from 23% to 33%, with an average of 28.9%. Medium stones (20–40 mm) made up 17% to 19% of the total, averaging 18.9%. Small stones (5–20 mm) ranged from 7% to 14%, averaging 11.1%. Sand particles (less than 5 mm) varied from 10% to 29%, averaging 18.8%. Within the gradation envelope, the coarsest gradation had a sand content of 10.3%, while the finest gradation contained 28.1% sand. The average fineness modulus of the sand was 1.44, classifying it as super-fine sand, with an average mud content of 15% (% of natural aggregate passing sieve no. 200; plasticity (PI) < 17)% [1,5].

### 2.2. Methods

#### 2.2.1. Phase 1: The Mix Proportion of CSGR

Based on the “double gradation and double strength” [1,5] principle proposed by Jinsheng Jia, the mixing material was prepared and controlled accordingly. The maximum coarse aggregate particle size was limited to 150 mm, while the cementitious material content was maintained at 80 kg/m^3^. The vibrating-compacted (VC) time at the mixer’s discharge was controlled between 2 and 8 s without admixture use. Detailed parameters are presented in Table 7, where schemes A1, B2, and C3 correspond to X1 aggregate (finest gradation with a sand ratio of 28.1%, and a distribution of extra-large stones/large stones/medium stones/small stones = 27.5:35.6:23.3:13.6), Y2 aggregate (average gradation with a sand ratio of 18.8%, and a distribution of extra-large stones:large stones:medium stones:small stones = 22.9:33.0:25.2:18.9) and Z3 aggregate (coarsest gradation with a sand ratio of 23.2%, and a distribution of extra-large stones:large stones:medium stones:small stones = 20.5:32.3:35.2:28.7), respectively. The gradation that produced Cemented Sand and Gravel (CSGR) laboratory compressive samples with a strength grade of C_180_6 (CSGR of compressive strength of 6 MPa after 180 days) at an 80–90% confidence rate was selected for the next phase.

#### 2.2.2. Phase 2: Grout Optimization

A standardized grout mixing procedure was implemented to assess the impact of chemical admixtures on the properties of grout. All grout formulations’ water-to-cement (w/c) ratio was fixed at 0.5–0.6 6. This w/c ratio was suggested considering previous experience with Grout Enriched Roller Grout Enriched CSGR and Compacted Concrete (GERCC). The chemical admixtures were initially mixed with water briefly before the gradual addition of cement. A paddle mixer, driven by a 1680 RPM drill, ensured consistent agitation during cement incorporation. Once all the cement had been added, the drill was operated at maximum speed for four (4) minutes to achieve a homogenous mix.

Upon completion of the four-minute mixing phase, the fluidity of each grout mixture was measured using the standard Marsh Funnel test according to ASTM D6910 [31], and the time required for 946 mL of grout to pass through the funnel was noticed and recorded. Following the Marsh Funnel test, the air content of each mix was measured by ASTM C231/C231M [32] using the Type-B pressure meter. A slight modification to the standard testing procedure was made to improve the accuracy of air content measurement by reducing foam presence in the pressure meter. Specifically, the grout was allowed to overflow the container slightly to eliminate excess foam, and a steel rod was used to strike off any remaining foam. This procedure was performed twice for each sample, followed by testing as per ASTM standards.

The remaining grout from each batch was allowed to sit undisturbed for 30 min. After this resting period, the grout was placed on a vibration table and subjected to one minute (60 s) of maximum intensity vibration, simulating the internal vibration of GECSGR during placement. The air content was then re-measured using the identical pressure method.

Six (6) Admixtures including organic air-entraining admixture (OAEA), synthetic AEA (SAEA), polycarboxylate-based water-reducing agent (WR), latex-based water-repelling and efflorescence–controlling admixture (LW), saline-based efflorescence–reducing and water-repelling admixture (SW), and a powdered water-resisting and superplasticizing admixture (PW). The water-repelling admixtures were specifically included to assess their influence on the stability of the air void system, as reducing permeability is a strategy to enhance freeze–thaw resistance. Initially, each admixture was evaluated independently at varying dosage levels, followed by combinations of admixtures to examine their interactions and effects on air matrix stability. A total of 37 grout formulations were tested, but 11 conditions of admixtures passed the above grout properties test using the methodology discussed above. The 11 with grout identity (ID) SL1 to SL11 are provided in Table 8 and proceeded for the GECSGR test [24].

#### 2.2.3. Phase 3: Determination of Grout Addition Rate

The grout addition rate was determined for grout formulated from Table 8 before application for both small and large-scale applications to determine the quantity of grout addition with respect to kg per m^3^ of CSGR. The performance indices for the selection were slump, compressive strength development, impermeability, and frost resistance. The mix for the grout is shown in Table 9.

#### 2.2.4. Phase 4: Small-Scale Laboratory GECSGR

In Phase 4, various fresh and hardened properties of CSGR, grout, and Grout-Enriched Vibrated CSGR (GECSGR) were analyzed. Tests included flow, air content, bleed, and compressive strength. Additionally, freeze–thaw resistance tests were conducted. Seven (7) chemical admixture combinations were evaluated for grout, including one grout mix without admixtures. These combinations were chosen based on Phase 2 and 3 results.

The CSGR mix was produced according to ACI 211.1 [33] and fine-tuned through trial mixes, targeting a Vebe time of not exceeding 25 s (SL-678-14) [10]. Two 150 mm by 150 mm cubes were cast per mix for 28-day compressive strength tests after wet screening to remove aggregate greater than 40 mm, with compaction simulated by filling in thirds and compacting using a Hilti TE 805 hammer drill, as per ASTM C1435 [34]. A 75 mm by 100 mm by 400 mm rectangular prism was also cast for freeze–thaw resistance tests, compacted with a steel block struck with a rubber mallet.

The grout was mixed using a ChemGrout CG-600 colloidal grout plant, with 42.6 kg of cement per batch. After four minutes of mixing, the grout was transferred for testing. Immediate and 30 min delayed tests were performed for flow (ASTM D6910) [31], specific gravity, and air content (ASTM C231) [32]. A Marsh Funnel test determined initial flow, while a mud balance measured specific gravity. Air content was checked using a Type B meter, and bleed stability was assessed using ASTM C1741 [35], where air was pumped into a filtration funnel at 172 kPa to collect bleed water.

After 30 min, the grout was placed on a shake table and tested again for specific gravity and air content. Simulated field conditions involved circulating grout through the plant, constricting the valve to reach 345 kPa, and re-testing the material.

Grout-enriched CSGR was produced by adding grout to the remaining 79.4 kg of CSGR mix and mixing in a batch mixer. A Vebe test and unit weight measurement were conducted, and two cubes were cast. Freeze–thaw samples were prepared and tested as per ASTM C666 [36]. These samples, three enriched with grout and one standard CSGR, were cured for 14 days in lime water, then weighed and tested for fundamental transverse frequency every 30 freeze–thaw cycles [24].

#### 2.2.5. Phase 5: Field Application of GECSGR

**Figure 3:**
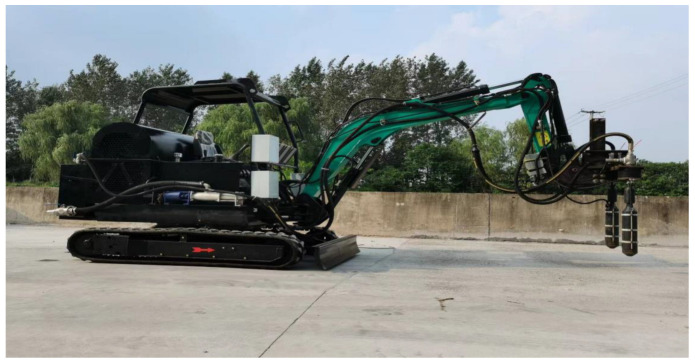
Newly developed 2-in-1 grouting and vibration equipment for GECSGR production.

The newly developed GECSGR was applied on a large scale for the time on the Tangba protection dam (the 2.76 km long Qianwei Dike in Sichuan Province of China). The usage of the newly developed 2-in-1 grouting addition and vibration equipment as a substitute for manual grouting and vibration helped control the post-vibration air content and improved the overall efficiency in the field application. The specialized equipment (shown in Figure 3) for this technique has a capacity of 12 m^2^/h [5].

#### 2.2.6. Limitations of the Research Methodology

The grout design and GECSGR technique did not consider silica fume in the grout preparation because silica fume increases the grout’s viscosity according to the ambient temperature, consequently creating difficulties with grout penetration into parent materials as reported from GERCC preparation from RCC. Therefore, special quality control measures with respect to the w/c ratio, slurry addition rate, and marsh time are essential to achieve the desirable outcome [21,24].

## 3. Results and Discussion

### 3.1. Results

#### 3.1.1. Phase 1: The Mix Proportion of CSGR

**Figure 4:**
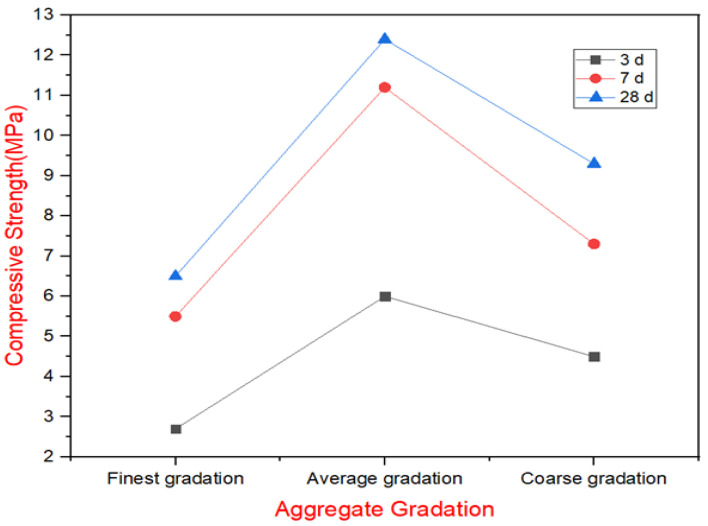
Compressive strength of CSGR using the varieties of aggregate gradations.

Table 10 and Figure 4 present the results of the compressive strength test of the CSGR materials. Since C_180_6 is the requirement as the strength class at an 80–90% confidence level for the production of the GECSGR in this project, the Y2 mix ratio corresponding to the medium gradation met this criterion and was selected to be added to the grout formulations for the GECSGR test.

#### 3.1.2. Phase 2: Grout Optimization

Mixtures without water-reducing admixtures had consistent flow times, ranging from 26 to 38 s, aligning with the target of 30 s. Mixtures with higher air content generally exhibited longer flow times, as increased air content reduces the unit weight and pressure of the grout, thereby slowing its velocity through the funnel.

**Figure 5:**
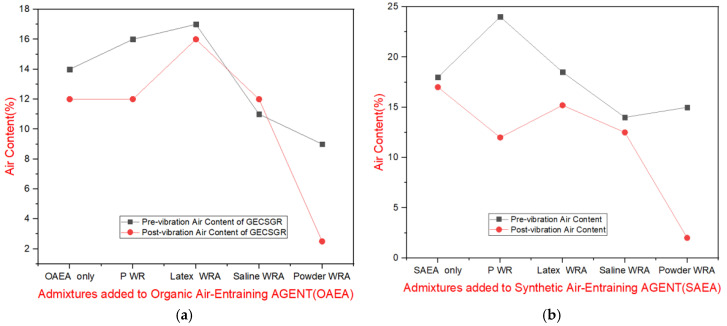
(**a**) Average air content for the grout containing organic air-entraining admixture; (**b**) average air content for grout containing synthetic air-entraining admixture.

For grout mixtures containing water reducers, flow times varied more until optimal dosages were determined. The final mix achieved flow times between 34 and 38 s, depending on the admixtures used. Figure 5a,b provide average air content results, highlighting key findings.

Figure 5 shows the air content of grout samples containing the organic air-entraining agent (OAEA) and Synthetic air-entraining agent (SAEA) at their optimal dosage, which produced prost-vibration air contents between 15 and 20%. This target was based on two factors: the foam stability during testing and calculations from previous studies. Figure 5a illustrates the effect of various chemical admixtures on the organic AEA, indicating that the latex-based water-repelling admixture enhanced the AEA’s stability, while the powder-based water-repelling admixture had a detrimental impact.

Similarly, Figure 5b presents the results for the synthetic AEA at its optimum dosage. The data revealed that water reducers and powder water-repelling admixtures decreased the stability of the air void system, whereas the latex-based admixture had minimal effect. Comparatively, the synthetic AEA was slightly more stable and maintained its performance at higher air contents. Consequently, in the next phases, more focus was placed on the synthetic AEA, and the powder-based water-repelling admixture was excluded due to its negative interaction with both AEAs [24].

#### 3.1.3. Phase 3: Determination of Grout Addition Rate

The slurry material needed optimal rheological properties to permit optimum penetration into the compacted cemented sand, gravel, and rock (CSGR). This will facilitate adequate compaction to meet the protection and anti-seepage design criteria. While a higher water/cement ratio was found to enhance rheological performance, it also increased the volume of slurry necessary to achieve the design specifications. To address this, Feng et al. [37] proposed a method that refines the slurry mix ratio by reducing the water/binder ratio while still ensuring efficient diffusion throughout the mixture and even distribution across the vibrating surface. The optimal slurry mix ratio and characteristics are detailed in Table 11. The rheological performance was regulated through marsh flow time, which was maintained between 26 and 31 s. Additionally, specific gravity, measured with a specific gravity meter, controlled the water/cement ratio during the mixing process [5].

A new formulation of cement slurry in Table 11 (based on experience and design practice) was selected as the grouting material and combined with the Y2 CSGR (C_180_6) from Table 10 for the slurry rate studies, six different slurry addition rates were tested: 5%, 6%, 8%, 10%, 12%, and 14%. The prepared grout-enriched CSGR mixtures were labeled as GEY2 (GECSGR prepared from the average gradation termed Y2 in Table 7; phase 1). Following the “Test Code for Hydraulic Concrete” (SL/T 352-2020) [38], the indoor mixing and forming method for GERCC was applied using a vibrating table. The slurry, according to Table 11, was added to the machine-mixed CSGR, followed by three rounds of manual mixing before specimen formation. Wet sieving was performed to remove particles larger than 40 mm, and the specimens were tested for slump and air content.

**Figure 6:**
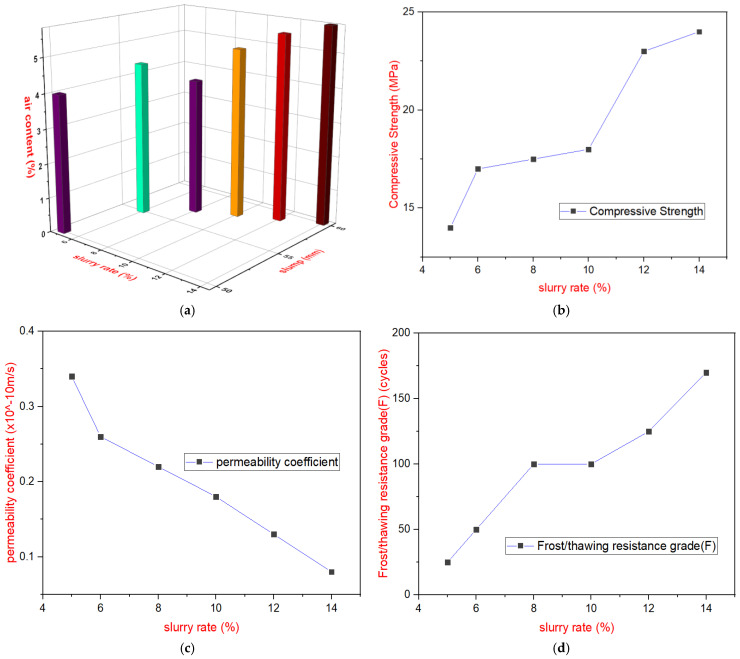
Effect of slurry rate on GECSGR performance. (**a**) Effect of slurry rate, slump, and air content. (**b**) Effect of slurry rate on compressive strength. (**c**) Effect of slurry rate on permeability coefficient. (**d**) Effect of slurry rate on frost/thawing resistance.

The test results are shown in Figure 6 above. Figure 6a reveals that the slump of the mixture after slurry addition ranges from 10 to 60 mm, while the air content ranges between 4% and 5.8%, indicating enhanced workability. Figure 6b also demonstrates that the compressive strength of the GECSGR increases with higher slurry rates. At an 8% slurry addition, with a total cement content of 176 kg/m^3^, the 90-day compressive strength exceeds 15 MPa for the GECSGR from GEY2, meeting design requirements for the field application (Qianwei dike) with an 80–90% confidence level. The impermeability grade reaches W8 (i.e., permeability coefficient of 0.26 × 10^−10^ m/s), and the frost resistance grade is F100, as shown in Figure 6c,d, making the material suitable for dams under 30 m in height (i.e., minimum impermeability grade W4 equivalent to permeability coefficient of 0.78 × 10^−10^ m/s).

At a 12% slurry addition rate, the cementitious content increases to 223 kg/m^3^, resulting in a 90-day compressive strength exceeding 20 MPa. The impermeability grade improves to W11 (equivalent to a permeability coefficient of 0.16 × 10^−10^ m/s), and the frost resistance grade rises to F125, fulfilling the design criteria for dams between 30 and 70 m height (minimum impermeability grade W6 or permeability coefficient of 0.42 × 10^−10^ m/s). However, at a 14% slurry addition rate, the cementitious material reaches 247 kg/m^3^, significantly raising the costs of GECSGR production due to the large amount of cement and admixture requirement and increasing the risk of crack development. As a result, this higher rate was eliminated [5].

#### 3.1.4. Phase 4: Small-Scale Laboratory GECSGR

During Phase 4, key properties of the grout, CSGR and GECSGR were measured. The flow times for the grout were recorded immediately after mixing and ranged between 26 and 38 s for most mixes. However, the grout containing a water-reducing admixture (WRA) had a significantly longer flow time. This extended flow time is likely due to the thixotropic behavior of the grout when a WRA is added, which increases flow resistance during measurement using the marsh funnel method.

Bleed measurements indicated substantial bleed across the grouts due to their high water-to-cement ratios, with an average of 84 mL. The lowest bleed volume, 39 mL, was observed in the grout with WRA, attributed to its reduced water-to-cement ratio.

**Figure 7:**
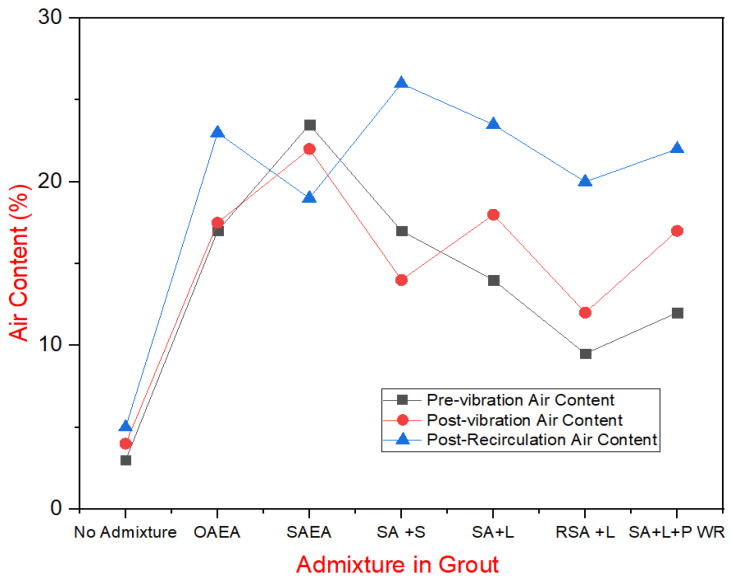
Air content in grout under varying admixture conditions.

Air content was measured at three stages: immediately after mixing, after 30 min of rest followed by one minute of vibration, and after 3 min of recirculation under pressure through the pump. These results are displayed in Figure 7, showcasing the behavior of the grout under varying admixture conditions.

Several key observations emerged from the Phase 4 air content data. Notably, the recirculation of grout through the pump did not negatively impact air content; instead, it often increased it. This rise in air content is likely due to the reintroduction of the pumped grout into the holding tank, where it splashed, incorporating more air. Additionally, since the pump drew material from the bottom of the tank, the lighter, more air-entrained grout at the top could have been used for recirculation testing, contributing to the higher air content readings. This also explains why grout samples that rested for 30 min before vibration generally had slightly higher air content.

The synthetic air-entraining agent (AEA) demonstrated superior performance, showing the highest and most consistent initial air contents, which indicates the mixture’s ability to remain homogeneous. This justified the use of synthetic AEA in evaluating other admixtures. Most other admixtures negatively affected the initial air content, with saline having the least impact. One sample, labeled “Reduced Synthetic AEA + Latex”, included a reduced dose of AEA to test if a high air content was necessary for achieving freeze–thaw resistance.

**Figure 8:**
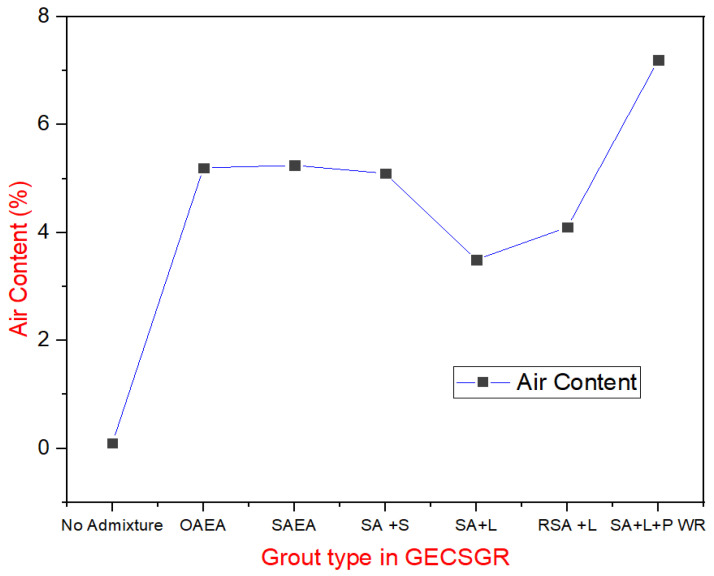
Air content in GECSGR under varying admixture conditions.

Air content measurements for the GECSGR, shown in Figure 8, corresponded well with expected values based on grout air content and dosage, achieving the target air content of 4–6% required for freeze–thaw resistance. The only exception was the grout with the water-reducing admixture, which had a much higher air content than expected. This could be due to the significantly higher flow time, indicating the grout was less fluid, potentially leading to poor compaction and artificially high air content measurements in the meter, which records all voids, including those from poor compaction.

**Figure 9:**
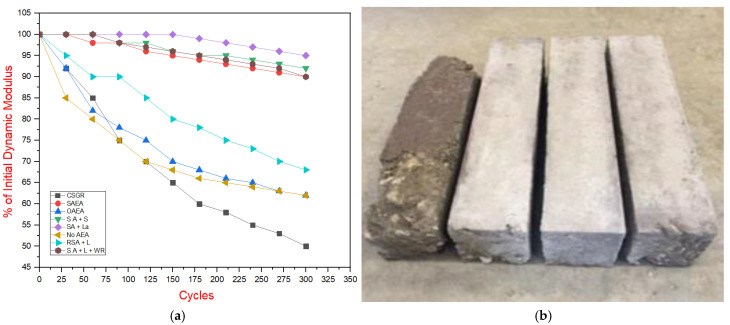
Freeze–thaw resistance of GECSGR. (**a**) Percentage of initial dynamic modulus of GECSGR. (**b**) Freeze–thaw samples after 300 cycles.

**Figure 10:**
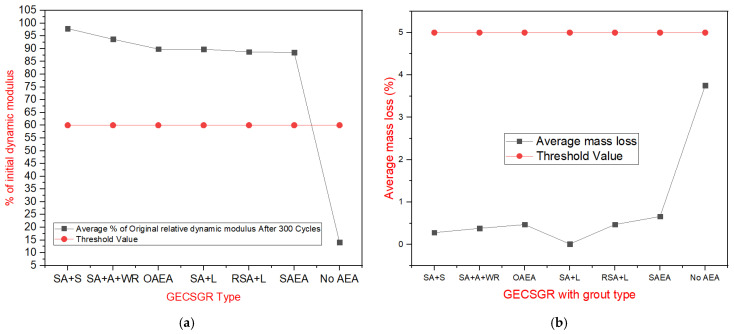
Average performance values of freeze–thaw resistance of GECSGR. (**a**) Average value of % of initial dynamic modulus of GECSGR. (**b**) Average value of mass loss of GECSGR after 300 cycles per admixture condition.

The freeze–thaw test results, as presented in the order of Table 12, Figure 9, and Table 13 and Figure 10, reveal important findings regarding the durability of the tested samples. Table 12 and Figure 9a illustrate the change in modulus of elasticity over the number of freeze–thaw cycles. The only samples to fail the test—defined as a drop in dynamic modulus below 60% of the original—were samples (shown in black) without admixtures. In contrast, all samples containing an air-entraining agent (AEA) retained at least 75% of their initial modulus.

Table 13 and Figure 10 summarize the average mass loss and percentage of the original dynamic modulus for each sample set, indicating the threshold values. The highest freeze–thaw resistance was observed in the synthetic AEA sample with the saline water-repelling admixture, which retained 97.9% of its initial modulus, which correlates well with Musselman et al. (2016) results of 96.8% on GERCC under similar conditions [24]. The remaining mixtures performed similarly well, all meeting satisfactory criteria for freeze–thaw resistance. Figure 9b visually compares the durability of samples with synthetic AEA and saline water-repelling admixtures against the corresponding plain CSGR (i.e., CSGR with no admixture) sample after 300 cycles, demonstrating a significant improvement in durability when air-entrained grout is used.

### 3.2. Discussion

#### 3.2.1. Phase 5: Large-Scale Field Application of GECSGR to Qianwei Dike

##### Project Requirements for Protect and Seepage Layer and Construction

The Qianwei Dike spans a total length of 2.76 km, featuring a crest elevation of 336.10 m, with a filling volume of 373,000 m^3^ of CSGR material [1]. During the design, the protection and seepage control layer made of GECSGR was required for the dam. Therefore, GECSGR of design specification C9015W6F50 was chosen after a series of laboratory and field tests meeting the project requirements (i.e., GECSGR sample of 90-day design compressive strength of 15 MPa, long-term permeability resistance grade of W6, equivalent to 0.24 × 10^−10^ m/s and could withstand long-term 50 cycles of frost/thawing with average relative dynamic modulus and average mass loss 60% and 5%, respectively).

**Figure 11:**
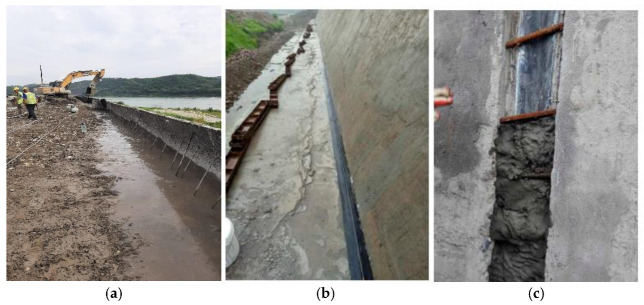
(**a**) Construction of the dam body and GECSGR; (**b**,**c**) upstream face T-shaped waterstop structure.

**Figure 12:**
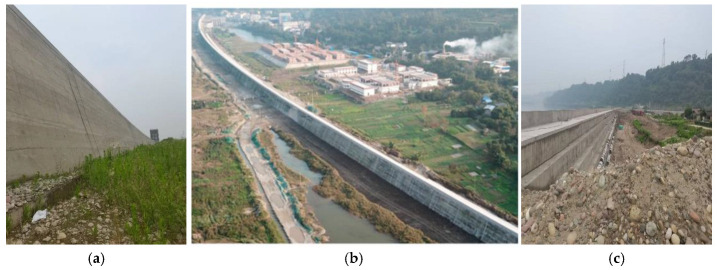
The completion of Qianwei Dike: (**a**) upstream face showing hardened GECSGR; (**b**) aerial view of the dike and nearby settlement after completing before impoundment; (**c**) downstream cascaded face of hardened GECSGR.

To create the protection and seepage control layers in both the upstream and downstream regions, GECSGR, with a slurry rate of 9%, was utilized. The construction was executed through sequential rolling and layer-by-layer techniques, resulting in a total of 20,867 m^3^ of GECSGR used. Figure 11 depicts the construction process of the body, the protection and seepage control layer, and the T-shaped waterstop simultaneously. The T-shaped surface water stop measures a total of 10,163 m and was installed and secured with ease, facilitating rapid construction and simplifying future replacement and repair efforts (see Figure 11b,c). Figure 12 represents the completion of the protection dam before impoundment [5].

##### Quality Test

The quality test results from the construction process are detailed in Table 14. The upstream protection and seepage control layer of the dam adheres to the design specifications of C9015W6F50 [5].

##### Performance Test

**Figure 13:**
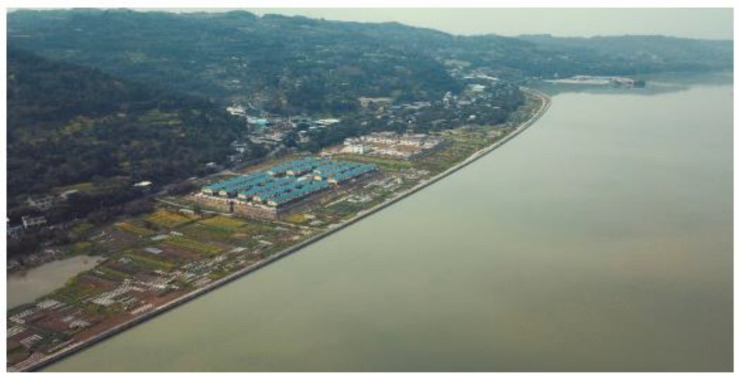
Qianwei Dike under successful full operation with the Tangba community downstream.

The first phase of water storage was completed from May 15 to 16, 2020, raising the water level from 326 m to 330 m. Following this, the second phase of water storage was finalized from September 15 to 16, 2021, resulting in an increase in the water level from 330 m to 335 m (see Figure 13). Water storage operations have been ongoing for nearly five (5) years. Importantly, optical fiber leakage measurement devices installed downstream of the transverse joint have shown no signs of leakage, demonstrating the dam’s excellent overall anti-seepage capabilities. Additionally, during the impoundment period, the levee withstood the catastrophic flood in 2020. Despite the severe impact of this disaster on Leshan and Qianwei, the dam effectively managed the flood, protecting the lives and property of over 11,000 residents and more than ten major enterprises in the Tangba Township. Overall, the dam’s safety and reliability have consistently met the expected standards [5].

##### Comparison of GECSGR, CVC, and GERCC in CSGRD Applications

GECSGR is created by grouting and vibrating directly onto pre-paved CSGR material. Unlike CVC, it plays a critical role in accommodating transitional deformations at the interface between the impermeable layer and the main body of the dam. This process enhances the continuity and integrity of the dam structure while reducing local damage and delamination caused by sudden changes in material properties. Additionally, GECSGR minimizes the diversity of materials needed at batching plants, thereby improving production efficiency and minimizing construction disruptions. Consequently, it is an ideal choice for future CSGRDs’ protection and seepage control layers as, aside from being a sustainable material, its unit production is also economical compared to CVC and GERCC (shown in Table 15) [5]. The environmental impacts of the three materials have been compared in Table 16, reinforcing the superiority of the adoption of GECSGR for CSGRD application [5].

This study explored the potential for producing air-entrained Grout-Enriched Vibrated CSGR and its application in low dams (dikes or levees). The research has reached a significant milestone; however, additional investigations are needed to thoroughly understand its long-term material performance in engineering applications, particularly for the exclusive use of GECSGR in medium-height dams (30–70 m) and higher dams. The successful implementation of air-entrained vibrated grout-enriched CSGR at the Qianwei Protection Dam provides a valuable case study for similar projects.

## 4. Conclusions

This paper introduces the successful development and application of air-entrained Grout-Enriched Vibrated CSGR (GECSGR) to the Qianwei protection low dam (dike). The following conclusions are therefore drawn:

1. Grout-enriched vibrated CSGR, if it meets the permeability and frost resistance grade of W12 (permeability coefficient of 0.13 × 10 m/s) and F300 (300 cycles of freeze and thawing with mass loss of <5% or relative dynamic modulus >60% of original), respectively, can be used for the construction of the protection and impermeable layer in CSGRD. These conditions are relaxed based on actual design and project requirements, as seen in the field application.
2. Employing a 223 kg/m^3^ cement slurry with a water-to-cement ratio of 0.5–0.6, an admixture of 2.23 kg/m^3^ and grouting rates of 8% and 12%, the C_180_4 or C_180_6 dry-hard cemented sand, gravel, and rock characterized by a Vibrating-Compacted (VC) of 25 s can be converted into GECSGR exhibiting a slump range of 10–60 mm, VC 2–8 s, corresponding to grades C9015W8F100 and C9020W11F125, respectively.
3. The organic and synthetic air-entraining admixtures successfully achieved air contents between 13 and 15% and 23–25% when used at their maximum recommended dosages during field applications. However, the synthetic air-entraining agent proved to be the most effective, exhibiting the most stable air content readings and providing superior freeze–thaw resistance when used with appropriate water-resisting admixtures. It can achieve an air content of 4–6%, making it the preferred choice for enhancing durability in GECSGR applications.
4. While the water-reducing admixture effectively reduced bleed and permeability, its inclusion led to significant variability in air content. As a result, the dosage of a water-reducing admixture should be controlled in air-entrained GECSGR for field applications due to its negative impact on maintaining consistent air content.
5. Both water-repelling and efflorescence–controlling admixtures improved the performance of air-entrained grout, with the saline-based admixture demonstrating superior freeze–thaw resistance. However, the powder-based water-resisting admixture negatively affected air entrainment, which required more future research; until then, the dosage should be controlled critically in air-entrained GECSGR.

The ongoing research focuses on optimizing CSGR paste and mortar margins (α and β) and grout mixing techniques, including variations in mud content, sand ratio, different water-reducing agents, and water-repelling powder admixtures. The goal is to establish precise limit criteria for enhancing the performance of CSGR and GECSGR. Also, to gain a comprehensive understanding of its long-term material performance and engineering applications related to CSGRD.

## Figures and Tables

**Table 1:** Cement quality test results.

Item	Density (g/cm^3^)	Fineness (%)	Specific Surface Area (cm^2^/g)	Standard Consistency (%)	Setting Time (min)		Stability
					Initial Setting	Final Setting	
Cement	3.16	6.6	3610	27.2	140	190	qualified
GB175-2020 requirements	-	-	-	-	≥45	≤600	qualified

**Table 2:** Cement chemical analysis.

Component	CaO	SiO_2_	Al_2_O_3_	Fe_2_O_3_	MgO	SO_3_	Na_2_O	K_2_O	LOI
Percentage (%)	60–67	17–25	3–8	0.5–6	0.5–4	1–3	0.1–1	0.1–1	0.5–3

**Table 3:** Cement mortar strength test results.

Item	Flexural Strength (MPa)		Compressive Strength (MPa)	
	3 d	28 d	3 d	28 d
Cement	5.7	7.8	30.6	49.5
GB175-2020 requirements	≥3.5	≥6.5	≥17.0	≥42.5

**Table 4:** Fly ash performance test results.

Item	Density (g/cm^3^)	Fineness (%)	Water Demand Ratio (%)	Activity Index (%)
Fly ash	2.47	20.0	98	70.6
GB/T1596-2017 Level II requirements	-	≤30	≤105	≥70

**Table 5:** Fly ash chemical analysis.

Component	SiO_2_	Al_2_O_3_	Fe_2_O_3_	CaO	MgO	SO_3_	Na_2_O	K_2_O	LOI
Percentage (%)	40–60	15–35	5–15	5–30	1–5	0.5–4	0.5–1.5	0.5–1.5	1–5

**Table 6:** Chemical and physical properties of admixtures.

Product	Chemical				Physical							
	pH	Cl^−^	Na_2_SO_4_	Alkali	Density	Bleeding Rate Ratio	Water Reduction	Difference in Setting (min)		Ratio of Compressive Strength		
		(%)	(%)	(%)	(g/cm^3^)	(%)	(%)	Initial	Final	3 d	7 d	28 d
OAEA	7.0–8.0	0.25	0.05	0.30	1.05	0.50	2	10	30	12	20	30
SAEA	7.0–8.0	0.5	0.05	0.30	1.10	0.50	2	10	30	13	21	30
WR	7.0–8.0	0.5	0.10	0.20	1.20	0.20	40	30	90	15	25	31
SW	7.0–8.0	0.5	0.10	0.30	1.10	0.30	5	10	30	13	22	33
LW	7.0–8.0	0.25	0.50	0.50	1.20	0.50	5	10	30	12	20	32
PW	7.0–8.0	0.5	0.10	0.30	0.80	0.20	30	30	60	15	25	32

**Table 7:** Mix proportion for C_180_6 CSGR production.

Scheme	Sand Ratio	W/Cm	VC	Quantity in kg for 1 m^3^ of CSGR				
	(%)		(s)	Water	Cement	Fly Ash	Coarse Aggregate	Fine Aggregate
A1(X1)	28.1	1.43	3.5	114	40	40	1649.39	644.61
B2(Y2)	18.0	1.06	6.0	85	40	40	1942.30	449.70
C3(Z3)	23.2	1.30	4.0	104	40	40	1845.33	556.64

**Table 8:** Grout performance evaluations.

Slurry ID	Slurry Assigned Acronym	Slurry Type	A Cubic Meter of Grout		Marsh Fluidity (s)	Density Kg/L
			Constituents (kg/m^3^)			
			Water	Cement		
SL1	No AEA	No AEA	211	223	26–33	1.95
SL2	OAEA	Organic AEA	211	223	26–33	1.88
SL3	SAEA	Synthetic AEA	211	223	26–33	1.85
SL4	WR	Water Reducer	211	223	34–38	1.92
SL5	L W	Latex WRA	211	223	26–33	1.89
SL6	S W	Saline WRA	211	223	26–30	1.90
SL7	P W	Powder WRA	211	223	27–34	1.87
SL8	SA+S	Synthetic AEA + Saline	211	223	26–31	1.84
SL9	SA + L + WR	Synthetic AEA + Latex + WR	211	223	34–38	1.83
SL10	SA + L	Synthetic AEA + Latex	211	223	28–31	1.82
SL11	RSA + L	Reduced Synthetic AEA + Latex	211	223	27–33	1.80

**Table 9:** Grout formulated from Table 7 for grout addition rate test.

Slurry ID	1 m^3^ of Slurry Material Dosage		
	Materials in kg/m^3^		
	Water	Cement	Admixture
S0750	112–211	223	2.23

**Table 10:** Lengshuihe auxiliary CSGRD.

Scheme	Compressive Strength (MPa)		
	3 d	7 d	28 d
Fine Gradation (X1)	2.7	5.5	6.5
Medium Gradation (Y2)	6.0	11.2	12.4
Coarsest Gradation (Z3)	4.5	7.3	9.3

**Table 11:** Slurry mix design and characteristics.

Slurry ID	1 m^3^ of Slurry Material Dosage			Marsh Fluidity (s)	Density Kg/L
	Materials in kg/m^3^				
	Water	Cement	Admixture		
S0750	112–211	223	2.23	26–31	1.884

**Table 12:** Frost and thawing resistance (dynamic modulus of elasticity).

	CSGR No Admixture	Synthetic AEA	Organic AEA	Synthetic AEA + Saline	Synthetic AEA + Latex	No AEA	Reduced Synthetic AEA + Latex	Synthetic AEA + Latex + WR
Cycles	CSGR	SAEA	OAEA	S A + S	SA + L	No AEA	RSA + L	S A + L + WR
	Relative Dynamic Modulus (% of the Original)							
0	100	100	100	100	100	100	100	100
30	92	100	92	100	100	85	95	100
60	85	98	82	100	100	80	90	100
90	75	98	78	98	100	75	90	98
120	70	96	75	98	100	70	85	97
150	65	95	70	96	100	68	80	96
180	60	94	68	95	99	66	78	95
210	58	93	66	95	98	65	75	94
240	55	92	65	94	97	64	73	93
270	53	91	63	93	96	63	70	92
300	50	90	62	92	95	62	68	90

**Table 13:** Average values of performance for frost and thawing resistance of GECSGR.

GECSGR	Acronym	Average % of Original Relative Dynamic Modulus After 300 Cycles	Average % of Mass Lost After 300 Cycles
Synthetic AEA + Saline	SA + S	97.9	0.28
Synthetic AEA + Latex + WR	SA + A + WR	93.7	0.38
Organic AEA	OAEA	89.9	0.47
Synthetic AEA + Latex	SA + L	89.8	0.01
Reduced Synthetic AEA + Latex	RSA + L	88.8	0.47
Synthetic AEA	SAEA	88.5	0.66
No AEA	No AEA	14.1	3.75

**Table 14:** Quality test results of the GECSGR [5].

Index and Value GECSGR		Grout-Enriched Vibrated CSGR [C9015W6F50]
Design Index	Impermeability grade	W6
	Compressive strength guarantee rate/%	80
	Compressive strength/MPa	15
	Frost resistance grade	F50
Measured Value	Impermeability grade	˃W6
	Compressive strength guarantee rate/%	88
	Minimum strength/MPa	11.8
	Average strength/MPa	18.6
	The standard deviation of compressive strength	3.8
	The qualified rate of frost resistance at design age/%	100

**Table 15:** Comparison of the cost of GECSGR, CVC, and GERCC in CSGR application [5].

Material	Quantity of Cementitious Materials (kg/m^3^)		Cost per Cubic Meter Production (USD)	Construction Technology	Remarks
	Cement	Fly Ash			
CVC	140	60	43.4	It requires vibration and grading of aggregate and has interference with CSGR rolling construction.	The average cost of aggregate is USD 12.6 per ton.
GERCC	151	36	41.02	It requires grouting, vibrating, and grading of aggregate and interferes with CSGR rolling construction.	The average cost of aggregate is USD 12.6 per ton.
GECSGR	151	36	19.88	It requires grouting, vibrating, and flexible aggregate grading and has less interference (high seamless transition) with CSGR rolling construction.	The average cost of aggregate is USD 2.8 per ton.

**Table 16:** Environmental impacts of CVC, GERCC, and GECSGR production.

Aspect	CVC	GERCC	GECSGR
1. Material Sourcing	⁻ Relies heavily on cement (typical usage: 300–400 kg/m^3^) and high-quality aggregates. ⁻ Significant environmental footprint from cement production.	⁻ Moderate cement content (100–150 kg/m^3^ for lean RCC and >150 kg/m^3^ for high-paste RCC). ⁻ Requires specific materials like fly ash or slag, which may involve transport emissions.	⁻ Uses locally available sand, gravel, and rock, reducing transport emissions. ⁻ Low cement content (40–80 kg/m^3^) minimizes CO_2_ emissions.
2. Energy Consumption	⁻ High energy use in cement production and transportation. ⁻ Mechanical mixing and curing facilities are required, increasing energy demands.	⁻ Lower energy requirements compared to CVC due to reduced cement usage. ⁻ Energy-intensive equipment like batching plants and vibratory rollers increase construction energy demand.	⁻ Energy efficiency due to lower cement usage and minimal processing of local materials. ⁻ Does not require advanced mixing or curing processes.
3. Carbon Emissions	⁻ High emissions from cement use and energy-intensive processes. ⁻ Estimated CO_2_ emissions: 300–400 kg CO_2_/m^3^.	⁻ Moderate emissions due to reduced cement content and efficient construction methods. ⁻ Estimated CO_2_ emissions: 150–250 kg CO_2_/m^3^.	⁻ Low emissions due to minimal cement use and local material reliance. ⁻ Estimated CO_2_ emissions: 100–200 kg CO_2_/m^3^ (depending on cement content).
4. Waste Generation	⁻ Generates more waste from processing aggregates and unused cementitious materials. ⁻ Disposal of unused materials can contribute to landfill usage.	⁻ Waste generation is moderate, primarily from unused concrete and fly ash. ⁻ Potential to reuse industrial by-products (e.g., fly ash).	⁻ Minimal waste as local materials reduce the need for extraction or processing by-products. ⁻ Can utilize onsite excavated material.
5. Watr ====Usage	⁻ High water consumption for mixing, curing, and cleaning equipment. ⁻ Wastewater may require treatment before disposal.	⁻ Moderate water usage due to lower cement content and reduced need for curing. ⁻ Cleaning and batching plants and equipment requires significant water.	⁻ Low water demand for mixing and curing compared to CVC and RCC. ⁻ Most water is used locally, reducing transport and waste.
6. Land Disturbance	⁻ High due to quarrying for aggregates and materials. ⁻ Large batching and curing sites increase land use footprint.	⁻ Moderate land impact due to material transport and batching plants. ⁻ Land use is higher than CSGR but lower than CVC.	⁻ Minimal disturbance due to the use of local materials. ⁻ Rock, sand, and gravel can often be sourced from site excavations or nearby areas.
7. Construction Impact	⁻ High noise and dust from mechanical equipment and transport. ⁻ Prolonged construction duration increases localized impact.	⁻ High noise and dust from vibratory rollers and concrete batching equipment. ⁻ Construction is faster, reducing long-term impacts.	⁻ Minimal noise and dust compared to RCC and CVC. ⁻ Less heavy machinery required.
8. Sustainability	⁻ Low sustainability due to heavy reliance on non-renewable cement and high energy demands. ⁻ High carbon footprint.	⁻ Moderately sustainable due to reduced cement and incorporating industrial by-products like fly ash or slag.	⁻ Highly sustainable due to the use of abundant local materials. ⁻ Reduced cement demand mitigates impact on raw material reserves.

## Data Availability

The original contributions presented in this study are included in the article. Further inquiries can be directed to the corresponding author.

## Abbreviations

The following abbreviations are used in the manuscript:

CSGR	Cemented Sand, Gravel, and Rock
CSGRD	Cemented Sand, Gravel, and Rock Dam
CMD	Cemented Material Dam
C_180_20	Rich-Mix (High Cementitious) CSGR with Compressive Strength of 20 MPa at 180 days
SL678-2014	Technical Guideline for Cemented Granular Materials issued by the Ministry of Water Resources of China in 2014
C9015W6F50	GECSGR of 15 MPa design Compressive strength after 90 days with permeability resistance grade of W6 (equivalent to 0.42 × 10^−10^ m/s) and withstand 50 cycles of frost/thawing
C9020W11F125	GECSGR of 20 MPa design Compressive strength after 90 days with permeability resistance grade of W11(equivalent to 0.16 × 10^−10^ m/s) and withstand 125 cycles of frost/thawing
VC	Vibrating-Compacted Value
CSG	Cemented Sand and Gravel
FSHD	Faced-Symmetrical Hardfill Dam
RCC	Roller Compacted Concrete
MSA	Maximum Size of Aggregate
CVC	Conventional Vibrated Concrete
ICOLD	International Committee on Large Dams
